# Time courses for pulmonary oxygen uptake and cardiovascular responses are similar during apnea in resting humans

**DOI:** 10.3389/fphys.2025.1524237

**Published:** 2025-03-13

**Authors:** Johan P. A. Andersson, Tim Bacanovic, Philip Chen, Angelica Lodin-Sundström, Amitava Halder, Gustav Persson, Mats H. Linér, Bodil Sjögreen

**Affiliations:** ^1^ Department of Experimental Medical Science, Lund University, Lund, Sweden; ^2^ Department of Health Sciences, Mid Sweden University, Sundsvall, Sweden; ^3^ Department of Biology, Lund University, Lund, Sweden

**Keywords:** apnea, face immersion, pulmonary gas exchange, hypoxia, hypercapnia, oxygen saturation, diving response

## Abstract

**Introduction:**

The pulmonary oxygen uptake is reduced during apnea, compared to eupneic baseline, preserving the pulmonary oxygen store. This study elucidates the time course for this reduction, comparing it to the time course for apnea-induced cardiovascular responses.

**Methods and results:**

Experiments involved two groups, performing apneas during rest, both without and with cold-water face immersion (A and AFI). The first group (n = 18) performed A and AFI of gradually increasing durations (from 15 to 120 s, order unknown to participant), allowing analysis of the time course for apneic pulmonary gas exchange. The second group (n = 18) performed A and AFI of identical durations (mean: 137 s), allowing analysis of cardiovascular and respiratory responses. The time course for pulmonary oxygen uptake was similar to the time courses for heart rate and cardiac output, i.e., following a brief increase from eupneic baseline during the initial 15 s of A and AFI, the oxygen uptake was gradually reduced during apnea, reaching a sub-eupneic level from 30 s of apnea and onwards. Changes were augmented during AFI compared to A. Observations confirmed that cardiovascular responses to apnea, including a reduced cardiac output, reduced peripheral blood flow, and most likely a peripheralization of blood volume, preserved the pulmonary oxygen store, while the peripheral venous oxygen stores were depleted to a greater extent.

**Conclusions:**

We conclude that the central, pulmonary oxygen store is preserved with augmented cardiovascular responses to apnea, at the expense of peripheral venous oxygen stores, with a time course similar to that of the cardiovascular responses.

## 1 Introduction

Apnea and cold-water immersions have been used as experimental tools for eliciting autonomic reflexes (Hayashi et al., 1997; Tio et al., 1999; Andersson et al., 2000). Both voluntary and involuntary episodes of respiratory arrest of varying durations are common during cardiac MRI investigations (Carlsson et al., 2004) and sleep apneas (Nathani et al., 2024). In addition, apnea is an essential component in freediving, an activity that has increased in popularity (Bain et al., 2018), especially since the foundation in 1992 of the nonprofit organization AIDA International for the development of the competitive aspects of the sport (Fitz-Clarke, 2018). Therefore, understanding the integrated respiratory and cardiovascular responses to apnea is of interest.

There are numerous studies that have provided insights into the time course for various cardiovascular responses during apnea in resting humans (Perini et al., 2008; Perini et al., 2010; Costalat et al., 2013; Fagoni et al., 2015; Sivieri et al., 2015; Taboni et al., 2019). An autonomic reflex that has received special attention is the vagally-induced bradycardia that is initiated by apnea (Perini et al., 2008; Costalat et al., 2013). In resting humans, the apneic heart rate (HR) typically displays three phases; an initial increase followed by a gradual decline until a final level of adjustment is established after approximately 30 s of apnea (Jung and Stolle, 1981; Perini et al., 2008; Perini et al., 2010). During apnea performed with a lung volume above the functional residual capacity and with relaxed respiratory muscles, the stroke volume (SV) will be reduced because of an impeded venous return (Ferrigno et al., 1986; 1987). This is explained by the high intrathoracic pressure, secondary to the inward recoil of the distended chest wall, that reduces cardiac preload. With the simultaneous reductions in HR and SV, the cardiac output (CO) is reduced during apnea (Ferrigno et al., 1986; 1987; Palada et al., 2007a; Perini et al., 2008). Together with the increase in parasympathetic stimulation of the heart, there is an increase in sympathetic nerve activity to vascular smooth muscle that induces a peripheral vasoconstriction (Leuenberger et al., 2001; Heusser et al., 2009). The associated increase in systemic blood pressure causes an increase in cardiac afterload, contributing to the reduced SV (Persson et al., 2023). In addition, the peripheral vasoconstriction will result in a redistribution of blood flow towards the brain and probably the myocardium. Limb blood flow is reduced (Sterba and Lundgren, 1988) while the blood flow in the carotid arteries and velocity in the middle cerebral artery increases (Pan et al., 1997; Palada et al., 2007b). In general, these cardiovascular changes follow the time course described above for the HR, with some variations (Persson et al., 2023). Combined, the cardiac and vascular responses are commonly referred to as the “diving response” (Gooden, 1994; Fitz-Clarke, 2018). The diving response can be initiated by apnea alone, but the response is enhanced by the combination of apnea and face immersion in cold water (Marsh et al., 1995; Andersson and Schagatay, 1998; Cherouveim et al., 2013). With cold-water face immersion, stimulation of thermoreceptors in the area innervated by the ophthalmic division of the trigeminal nerve is of special importance for the augmented response (Khurana et al., 1980).

In contrast to the preponderance of data on the apneic cardiovascular responses, there is a lack of data on the time course for the pulmonary gas exchange during apnea. Nevertheless, the time-averaged pulmonary gas exchange for the entire duration of apnea is reduced compared to the eupneic control level, at the least for apneas of a duration longer than 60 s (Linér and Linnarsson, 1994; Wein et al., 2007; Andersson et al., 2008). With the reduced pulmonary O_2_ uptake and reduced peripheral blood flow, there is a gradual reduction in peripheral tissue and venous blood O_2_ levels (Valic et al., 2006; Schagatay et al., 2007; Bouten et al., 2020). The reduction in pulmonary gas exchange is predominantly explained by the circulatory adjustments during apnea, of which the reduction in CO and thus pulmonary perfusion are of particular importance (Linér et al., 1993; Linér and Linnarsson, 1994; Lindholm and Linnarsson, 2002; Andersson et al., 2004). To what extent the time course for the pulmonary gas exchange during apnea follows the time course for the cardiovascular responses is largely unexplored. Because of the close relation between pulmonary gas exchange and CO (Linér and Linnarsson, 1994), it is probable that the apneic pulmonary gas exchange displays a time course similar to that for the changes in apneic CO. However, the time course for changes in pulmonary gas exchange, together with cardiovascular responses, has not been specifically addressed in earlier studies concerning apneas in resting humans.

The present study is based on experiments involving two groups of participants, performing apneas both with and without cold-water face immersion. In the first group, apneas of varying durations were investigated which allowed analysis of the pulmonary O_2_ uptake and CO_2_ elimination during specified apneic periods, enabling us to elucidate the time course of the pulmonary gas exchange during apnea with and without face immersion. In the second group, for which we had access to additional instruments for recordings of cardiovascular and respiratory changes induced by apnea, we could elucidate the integrated physiological responses to apneas with and without face immersion. We hypothesized that there would be a gradual decrease in pulmonary O_2_ uptake during apnea compared to the eupneic control, which would reflect the time course of the cardiovascular responses to apnea, and that the physiological changes would be augmented by face immersion in cold water.

## 2 Material and methods

The study involved two groups of participants, with both groups performing apneas either with or without cold-water face immersion. The two groups and their respective experimental protocols will henceforth be referred to as group I and group II.

### 2.1 Ethics approval statement

All trials were conducted in conformity with the principles of the Declaration of Helsinki. The protocol for group I of the study was reviewed and approved by the research ethics committee at Lund University (LU 25-01), which was the relevant governing authority at the time of trials. The protocol for group II was reviewed and approved by the Swedish Ethical Review Authority (2022-04298), the current governing authority in Sweden. With the recruitment of participants, they were provided written information about, e.g., the procedures, potential risks, and handling of data. At the laboratory, after verbal clarification of test procedures and potential risks involved, the participants provided their oral and written informed consent to participate in this study. Exclusion criteria were age below 18 or above 55 years, any known acute or chronic disease, use of medications (except for contraceptives), and pregnancy. Anomalies in blood pressure, lung function, or electrocardiogram (ECG), measured and evaluated at the beginning of each trial, would lead to discontinuation of the trial.

### 2.2 Subjects

Healthy volunteers were recruited for the study. The number of recruited participants was based on the expected variance of results, relating to previous, similar studies (Andersson et al., 2008; Perini et al., 2010; Taboni et al., 2019). For group I, eighteen participants volunteered, and another eighteen participants volunteered for group II. Participants for group I were recruited among breath-hold divers (n = 8), under-water rugby players (n = 7), scuba divers (n = 2), or subjects having performed long apneas in previous studies at our laboratory (n = 1), i.e., all participants had some to extensive previous experience of apnea. Of the participants in group II, eleven were either swimmers or breath-hold divers with some to extensive previous experience of apnea, while seven were not performing apnea regularly. The characteristics of both groups are presented in Table 1. Most participants were physically active, and besides diving activities, their self-reported physical training averaged 4 h/week, without a difference between groups. All participants were non-smokers. The participants were instructed to arrive at the laboratory after at least 2 h without any heavy meal or caffeine-containing beverages, with only light physical activity being performed within 12 h of the trial.

**TABLE 1:** Characteristics of participants in group I and group II.

	Group I	Group II	
Sex (F/M)	18 M	3 F/15 M	
Age (yr)	24 (3), 19–29	29 (11), 20–53	n.s.
Height (cm)	183 (4), 176–190	178 (8), 162–192	*p* = 0.03
Body mass (kg)	78 (7), 64–96	75 (11), 58–91	n.s.
Vital capacity (L)	6.0 (0.9), 4.8–7.4	5.6 (1.0), 4.2–7.0	n.s.
Residual volume (L)	1.6 (0.3), 1.1–2.4	1.6 (0.4) 1.0–2.7	n.s.

Values are means (SD) and range, except for sex which are number of participants (F, female; M, male); n.s., no significance.

### 2.3 Protocol

When participants arrived at the laboratory [air temperature 21.8°C (0.8), ambient pressure 755 mmHg (10), relative humidity 35.8% (9.8)], they received verbal information about the experimental protocol and the equipment that was to be used. All participants were given the opportunity to ask any questions about the procedures before signing the informed consent form for participation in the study. The participant completed a health questionnaire, after which the height and weight of the participant were measured. Blood pressure was measured in the seated position, while spirometry was performed with the participant in the standing position. Thereafter, the participant assumed a supine position on a mattress, and an ECG was recorded. Blood pressure, lung function, and ECG were assessed for anomalies.

After the ECG had been evaluated and the subject had been cleared for continued participation, the participant assumed a prone position on the mattress, and this position was maintained for the remainder of the test. The participant’s head rested on a removable pillow on top of a container used for cold-water face immersions, and the forearms rested on both sides of the container at the level of the heart (*cf.* Figure 1 in Schagatay and Andersson, 1998). During the experiments, the water temperature was maintained at 9°C–11°C. The vital capacity in this position was measured, and the volume corresponding to 85% of the vital capacity in the prone position was calculated. The probes of the instruments used for recordings of cardiovascular variables and oxygen saturations (*cf.* section 2.4) were attached, and the participant was reminded about some of the details in the protocol that was dependent on the participant’s correct performance (e.g., procedures just before and after apneas). The participant was told to relax and avoid voluntary hyperventilation, and to avoid Valsalva and Mueller maneuvers during apneas. After stable cardiovascular data were observed, recordings began and were continuously run until after the end of the last apnea in the protocol.

Group I: From the time recordings began, after 3 min of rest, the subject repeatedly performed apneas either without face immersion or with the face immersed into the cold water, alternating between conditions. The apneas lasted either 15, 30, 45, 60, 90, or 120 s in an order that was unknown to the participant. Each apneic time and condition were performed twice, adding up to a total of 24 apneas. The participant was unaware about the apneic duration that was to be performed and thus had to approach the performance as if each apnea was supposed to be sustained to the individual maximal duration. The apneas were separated by 3-min breathing pauses, during which the participant was breathing through a mouthpiece from an open-circuit spirometry system, with a nose-clip attached during both apnea and eupnea. Apneas were initiated after a countdown from one of the experimenters. During the last 10-s countdown, the participant exhaled to the residual volume through the open-circuit spirometry mouthpiece and inhaled, from a pre-filled rubber bladder, a volume of air equal to 85% of the individual prone vital capacity. During apnea without face immersion, the face was held right above the water surface, whereas during apnea with face immersion, the entire face, including the chin and forehead, was immersed. Without providing any continuous time cues during apnea, the experimenter notified the participant just before the intended end of apnea, so that, on command from the experimenter, the participant could end the apnea with a maximal exhalation through the open-circuit spirometry mouthpiece.

Group II: Each participant in this group performed a total of six apneas. The first two were performed to the individual maximal duration, one of these with and one without face immersion in cold water (alternating the starting order among participants). No time cues were provided by the experimenters during these maximal-duration apneas. Based on the individual maximal duration, an individual submaximal breath-holding time was set for the remaining four apneas; the median difference between individual maximal and sub-maximal apnea times was 16 s. The four sub-maximal apneas also alternated between either apnea with face immersion or apnea without face immersion. During the sub-maximal apneas, the participant was given time cues by the experimenter. The sub-maximal apneas were the ones used for subsequent analysis. All apneas were separated by 5-min breathing pauses, except for the last maximal and first sub-maximal apnea that were separated by a 10-min period. A nose-clip was attached when 30 s remained before apnea, just before the participant began breathing through the open-circuit spirometry mouthpiece. The initiation and end of apneas in group II followed the same procedure as in group I, including inhaling 85% of prone vital capacity from the residual volume to initiate apnea, and ending the apnea with a maximal exhalation through the open-circuit spirometry mouthpiece.

In both group I and group II, beginning 5 minutes after the last apnea, the residual volume in the prone position was measured with a nitrogen-dilution technique (Rahn et al., 1949). In short, after a maximal exhalation to residual volume the participant rebreathed five times through the open-circuit spirometry mouthpiece, which at this point was connected to a rubber bladder initially containing 3 L of 100% O_2_. The dilution of inert gas in this closed system, measured in the third exhalation, was used for calculation of the residual volume.

### 2.4 Measurements and data collection

A wall-mounted height measurer and an electric scale (BF214, Omron Healthcare Europe, Hoofddorp, Netherlands) were used to measure height and weight, respectively. Pre-trial blood pressure in the seated, resting position, was measured using an automatic sphygmomanometer (Boso-medicus, Bosch + Sohn GMBH, Jungingen, Germany). A hand-held spirometer (Micro Plus, Micro Medical Ltd., Rochester, England) was used for spirometry measurements in both the standing and prone positions. Because glossopharyngeal insufflation, a technique commonly used by competitive freedivers to increase the volume of air in the lungs, is associated with potential adverse effects (Andersson et al., 2009; Chung et al., 2010; Linér and Andersson, 2010), this technique was not allowed during spirometry or the rest of the protocol. An ECG-monitor (group I: Cardisuny 501, Fukuda ME Kogoyo Co., Tokyo, Japan; group II: Cardiovit AT-1 G2, Schiller, Doral, FL, United States) was used for recording the ECG prior to further testing.

During the trials, respiratory flow and expiratory O_2_ and CO_2_ fractions were recorded using an open-circuit spirometry system (group I: CPX/D Cardiopulmonary Exercise System, Medical Graphics, Minneapolis, MN, United States; group II: Ergocard Professional, Medisoft, Sorinnes, Belgium). The open-circuit spirometry systems were calibrated using a 3-L syringe (Hans Rudolph, Shawnee, KS, United States) and certified gases (AGA Gas, Lidingö, Sweden, or Linde Gas, Solna, Sweden) prior to the start of each trial. Temperature, barometric pressure, and humidity were measured in the laboratory just prior to each experimental session, and temperature in both the ambient air and the water container used for face immersions were noted just before each apnea. From the recorded expired gas fractions and ambient pressure, end-tidal partial pressures of O_2_ and CO_2_ (P_ET_O_2_ and P_ET_CO_2_) were calculated.

In group I, the HR was recorded continuously with a HR monitor (Polar Vantage NV, Polar Electro Oy, Kempele, Finland). In group II, HR, SV, CO, total peripheral resistance (TPR), and arterial blood pressures were recorded continuously using a finger photoplethysmograph (Finapres NOVA, Finapres Medical Systems BV, Enschede, Netherlands). The Finapres NOVA monitoring system records finger arterial pressure using a finger cuff with a built-in photoplethysmograph (Bogert and van Lieshout, 2005). The finger arterial pressure recording is calibrated using the Physiocal algorithm, and the finger pressure is reconstructed into brachial arterial pressure, applying waveform filtering and level correction, which in turn is calibrated with a brachial blood pressure cuff. The finger cuff and the brachial cuff were unilaterally placed on the left middle finger and over the left brachial artery, respectively. The Finapres NOVA uses the Modelflow® algorithm to calculate cardiovascular variables, such as SV, CO, and TPR, from the recorded finger arterial pressure (Wesseling et al., 1993; Bogert and van Lieshout, 2005). The reconstructed arterial brachial pressure was recorded during the trials.

In group II, the deltoid muscle oxygen saturation (SmO_2_) was recorded, every 4 seconds, using a regional oximeter (Nonin SenSmart Model X-100 Universal Oximetry System, Nonin Medical, Plymouth, MN, United States), with an adhesive probe (SenSmart Equanox 8204CA rSO_2_ sensor, Nonin Medical, Plymouth, MN, United States) attached to the skin above the left deltoid muscle, 5 cm below the acromion. The arterial hemoglobin oxygen saturation (SaO_2_) was recorded continuously using a finger pulse oximeter (Biox 3700e, Ohmeda, Madison, WI, United States), with the probe placed on the left index finger.

The recordings of cardiovascular and respiratory variables began prior to the first test and continued until after the end of the last test using a data acquisition system (MP100, BIOPAC Systems, Goleta, CA, United States) connected to personal computers, and the data was stored for later analysis.

### 2.5 Data analysis

Group I: For each participant, baseline eupneic P_ET_O_2_ and P_ET_CO_2_, pulmonary O_2_ uptake and CO_2_ elimination, as well as HR were calculated as mean values from the period 90–30 s prior to all apneas. P_ET_O_2_ and P_ET_CO_2_ were determined from the last expiration before apnea and the first expiration that ended apnea. In addition to the determinations of P_ET_O_2_ and P_ET_CO_2_, theoretical partial pressures of O_2_ and CO_2_ of the pulmonary gas at the beginning of apnea (t = 0) were calculated by combining the composition of the gas of the residual volume and the inhaled ambient air (85% of prone vital capacity). Pulmonary gas exchanges during apneas were calculated from the differences between volumes of O_2_ and CO_2_ in the lungs at the beginning of and at the end of apnea, representing alveolo-capillary O_2_ and CO_2_ transfer. The volume of O_2_ in the lungs at the beginning of apnea was calculated by adding the volume of O_2_ in the rubber bladder to the volume of O_2_ in the residual volume. The latter was obtained using the measured end-tidal fraction of O_2_ in the last, maximal expiration prior to each apnea. The same calculations were done for the volumes of CO_2_ and inert gases. For determination of the lung volume at the end of apnea, it was assumed that the volume of inert gases in the lungs was constant during apnea (Hong et al., 1971; Linér et al., 1993). The O_2_ and CO_2_ volumes in the lungs at the end of apnea were subsequently calculated using the end-apnea lung volume and the end-tidal fractions of the maximal expiration following each apnea (Andersson et al., 2004). The difference in pulmonary gas volumes between apneas of increasing durations was used to calculate the gas exchange during the different apneic periods, i.e., 0–15, 15–30, 30–45, 45–60, 60–90, and 90–120 s. For determinations of the breath-holding time, the recorded tracings of expiratory O_2_ and CO_2_ fractions were used (Girardi et al., 2023), and the times required for inhalation from and exhalation to residual volume were included. Apneic values for HR were calculated as means for each of the above specified apneic periods using the 2-min apneas without or with face immersion. For all variables, the different apneic periods’ mean values were compared to the baseline, eupneic value. Also, apneas without and with face immersion were compared.

Group II: For each participant, baseline eupneic mean values for cardiovascular variables (HR, SV, CO, TPR, and arterial blood pressures), SmO_2_, and SaO_2_ were calculated from the period 90–30 s prior to the sub-maximal duration apneas. The apneic mean values for cardiovascular variables and SmO_2_ were calculated from the period 30–120 s into each sub-maximal duration apnea. In addition, for the cardiovascular variables, mean values for the apneic periods 0–15, 15–30, 30–45, 45–60, 60–90, and 90–120 s were calculated. For SaO_2_, the nadir in the 0–60 s post-apnea period was determined. P_ET_O_2_ and P_ET_CO_2_ were determined from the last expiration before apnea and the first expiration that ended apnea. As in group I, the pulmonary gas exchanges during apneas were calculated from the differences between volumes of O_2_ and CO_2_ in the lungs at the beginning of and at the end of apneas.

For each participant, individual mean values from the two apneas of each type and duration were calculated. IBM SPSS Statistics, Version 29.0.2.0 (IBM Corp, Armonk, NY) was used to perform statistical analysis. Data was checked for normal distribution, using the Shapiro-Wilk test, before further statistical tests were performed. For analysis of changes during apneas compared to baseline, one-way repeated measures analysis of variance with Bonferroni-corrected pairwise comparisons were used for data following a normal distribution, whereas Friedman tests with Bonferroni-corrected Wilcoxon signed rank test were used for data not following a normal distribution. For analysis of differences between apneas without and apneas with face immersion paired samples, two-tailed *t*-tests were used for data following a normal distribution, and related-samples Wilcoxon signed ranks tests were used for data not following a normal distribution. Data from participants of group I were compared to data from participants of group II using independent samples, two-tailed *t*-tests. The level used for accepting significance was *p* < 0.05. Values reported in the text are means (SD), unless otherwise stated.

## 3 Results

### 3.1 Group I

All participants completed all apneas to the intended durations. Compared to baseline, the P_ET_O_2_ was higher and the P_ET_CO_2_ was lower in the last expiration before apnea (Table 2). The pulmonary PO_2_ became even higher and pulmonary PCO_2_ even lower with the addition of the large volume of ambient air to the residual volume, i.e., after inhalation of air from the rubber bladder. Thereafter, during apnea, the P_ET_O_2_ and P_ET_CO_2_, measured in the first post-apneic expiration, decreased and increased, respectively, with time during both apnea without and with face immersion. For the two longest apneic durations, the P_ET_O_2_ was lower after apnea without face immersion than after apnea with face immersion (*p* < 0.01), while the P_ET_CO_2_ did not differ between apneic conditions.

**TABLE 2:** End-tidal partial pressures of oxygen and carbon dioxide before and at the end of apneas with successively increased breath-holding times. Apneas were performed either without or with face immersion in cold water.

Time	P_ET_O_2_ (mmHg)		P_ET_CO_2_ (mmHg)	
	A	AFI	A	AFI
Baseline	99.3 (7.0)		39.3 (3.9)	
Pre	111.9 (6.3)^***^		34.4 (3.8)^***^	
0 s	140.6 (1.6)^***^		7.9 (1.2)^***^	
15 s	115.6 (5.3)^***^	116.2 (5.1)^***^	37.6 (3.4)	37.4 (2.7)
30 s	108.1 (6.5)^*^	109.2 (5.7)^**^	39.5 (4.1)	39.2 (3.6)
45 s	102.0 (7.2)	102.3 (6.7)	41.1 (4.0)	41.2 (3.8)^*^
60 s	94.8 (7.9)	96.6 (8.4)	42.7 (3.8)^***^	42.3 (4.0)^***^
90 s	82.0 (10.1)^***^	84.2 (10.7)^**,^^††^	45.5 (3.5)^***^	44.9 (4.4)^***^
120 s	69.8 (11.9)^***^	72.3 (12.3)^***,^^††^	48.4 (3.4)^***^	48.0 (4.4)^***^

Values are means (SD), n = 18 (group I). Baseline values were collected 90–30 s pre-apnea. Pre-values were obtained from the final, maximal expirations before apneas. Values for 0 s are theoretical partial pressures as described in “Data analysis”. The values for different end-apnea times were obtained from the maximal expirations terminating apneas. A, apnea without face immersion; AFI, apnea with face immersion; P_ET_O_2_, End-tidal partial pressure of oxygen; P_ET_CO_2_, End-tidal partial pressure of carbon dioxide.

*p < 0.05, **p < 0.01, and ***p < 0.001 compared to baseline. ^††^ *p* < 0.01 compared to A.

**FIGURE 1:**
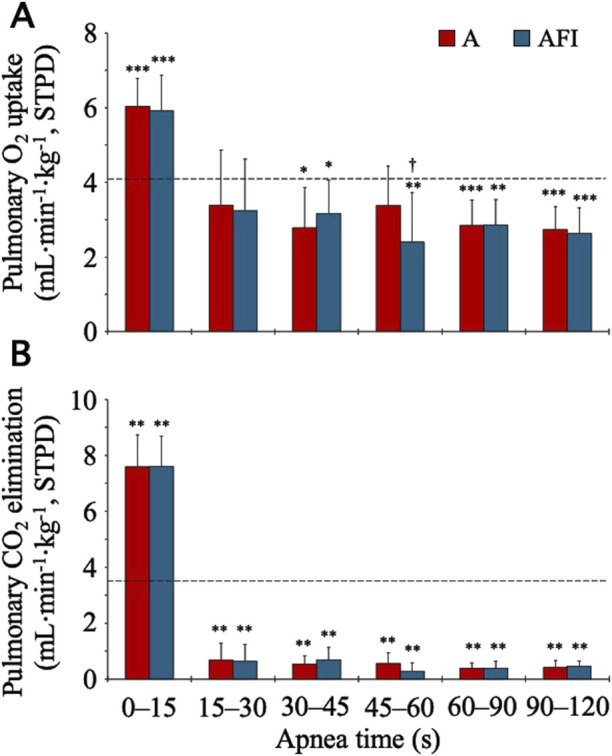
Pulmonary gas exchange during apnea, either without (A) or with face immersion (AFI) in cold water (n = 18, group I). Each bar represents the mean (SD) pulmonary O_2_ uptake and CO_2_ elimination normalized per kilogram of body mass [Panels **(A, B)**, respectively] during the specified apneic periods. The horizontal, dashed lines represent the eupneic, baseline pulmonary O_2_ uptake and CO_2_ elimination. ^*^ *p*< 0.05, ^**^ *p*< 0.01, and ^***^ *p*< 0.001 compared to baseline. ^†^ *p*< 0.05 compared to apnea without face immersion.

The baseline, eupneic pulmonary O_2_ uptake before apnea was 4.07 (0.69) mL⋅min^-1^⋅kg^-1^ and the CO_2_ elimination was 3.49 (0.90) mL⋅min^-1^⋅kg^-1^. During apneas, both the O_2_ and CO_2_ exchanges were increased above baseline for the initial 0–15 s (Figure 1). After this increase, the pulmonary O_2_ uptake was gradually reduced so that after 30 s, it was reduced compared to eupneic baseline. The time-averaged O_2_ uptake during the entire 2-min apnea without face immersion, 3.57 (0.48) mL⋅min^-1^⋅kg^-1^, was lower than baseline (*p* = 0.02 vs. baseline). The O_2_ uptake was even further reduced during the entire 2-min apnea with face immersion, to 3.42 (0.48) mL⋅min^-1^⋅kg^-1^ (*p* = 0.002 vs. baseline; *p* = 0.006 vs. apnea without face immersion). During the period 30–120 s into apnea without and with face immersion, when the reduction in gas exchange had stabilized, the O_2_ uptake was 71 (14) % and 68 (12) % of baseline O_2_ uptake, respectively. After the initial increase, the pulmonary CO_2_ elimination displayed a fast reduction from eupneic baseline, being evident after 15 s of apnea, and then stabilizing at these reduced levels. The time-averaged CO_2_ elimination was reduced to 1.84 (0.24) and 1.82 (0.25) mL⋅min^-1^⋅kg^-1^ during the entire 2-min apneas without and with face immersion, respectively (*p* < 0.001 vs. baseline; NS between apneic conditions).

**FIGURE 2:**
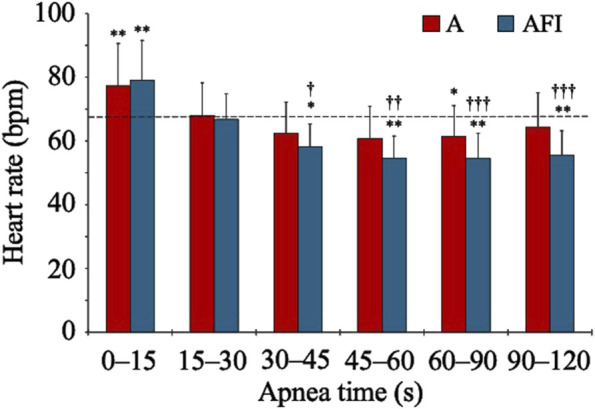
Heart rate during apnea, either without (A) or with face immersion (AFI) in cold water (n = 18, group I). Each bar represents the mean (SD) heart rate during the specified apneic periods. The horizontal, dashed lines represent the baseline heart rate. ^*^ *p* < 0.05, ^**^ *p* < 0.01, and ^***^ *p* < 0.001 compared to baseline. ^†^ *p* < 0.05, ^††^ *p* < 0.01, and ^†††^ *p* < 0.001 compared to apnea without face immersion.

The baseline, eupneic HR before apnea was 67.6 (10.3) bpm. During apneas, the HR increased above baseline for the initial 0–15 s (Figure 2). Thereafter, the HR gradually decreased, especially during apnea with face immersion, and reached a stable level below the eupneic baseline after 30 s of apnea. During the period 30–120 s into apnea, the HR was reduced from baseline in both apnea without face immersion (*p* = 0.028 vs. baseline) and apnea with face immersion (*p* < 0.001 vs. baseline, *p* < 0.001 between apneic conditions). During this period, the average HR was 94 (13) % and 82 (10) % of baseline HR, respectively.

### 3.2 Group II

The mean maximal apnea time was 156 (39) s, with a range among participants of 108–237 s. Based on the individual maximal apnea time, the sub-maximal apnea time was individually determined to be set at an average of 137 (31) s, with a range of 90–195 s. All participants completed all sub-maximal apneas to the intended duration.

Just as in group I, apnea caused a reduction in P_ET_O_2_, measured in the first post-apneic expiration, in group II (Table 3), and the reduction was greater during apnea without face immersion in cold water (*p* = 0.010). This was reflected by a lower pulmonary O_2_ uptake during apnea with face immersion than during apnea without face immersion (*p* = 0.012). The P_ET_CO_2_ was increased by apnea, without a difference between apneic conditions. Likewise, there was no difference in apneic pulmonary CO_2_ elimination between apnea with or without face immersion.

**TABLE 3:** End-tidal gas pressures and pulmonary gas exchange in relation to equal, sub-maximal duration apneas performed either without or with face immersion in cold water.

	P_ET_O_2_ (mmHg)		P_ET_CO_2_ (mmHg)		$\dot{V}$ O_2_/kg (mL⋅min^-1^⋅kg^-1^, STPD)	$\dot{V}$ CO_2_/kg (mL⋅min^-1^⋅kg^-1^, STPD)
	Pre	Post	Pre	Post		
A	119.7 (7.8)	59.4 (14.9)^***^	31.5 (3.9)	46.9 (3.4)^***^	3.68 (0.65)	1.57 (0.41)
AFI		62.2 (14.7)^***,^^††^		46.8 (3.3)^***^	3.56 (0.66)^†^	1.58 (0.41)

*Values are means (SD), n = 18 (group II). Pre-values were obtained from the final, maximal expirations before apneas. Post-values were obtained from the maximal expirations terminating apneas. Pulmonary gas exchanges are time averaged for the entire apneic durations. A, apnea without face immersion; AFI, apnea with face immersion; P*_*ET*_ *O*_*2*_ *, End-tidal partial pressure of oxygen; P*_*ET*_ *CO*_*2*_ *, End-tidal partial pressure of carbon dioxide;* $\dot{V}$ *O*_*2*_ */kg, Pulmonary oxygen uptake normalized per kilogram of body mass;* $\dot{V}$ *CO*_*2*_ */kg, Pulmonary carbon dioxide elimination normalized per kilogram of body mass.*

***p < 0.001 compared to Pre. ^†^ *p* < 0.05 and ^††^ *p* < 0.01 compared to A.

**FIGURE 3:**
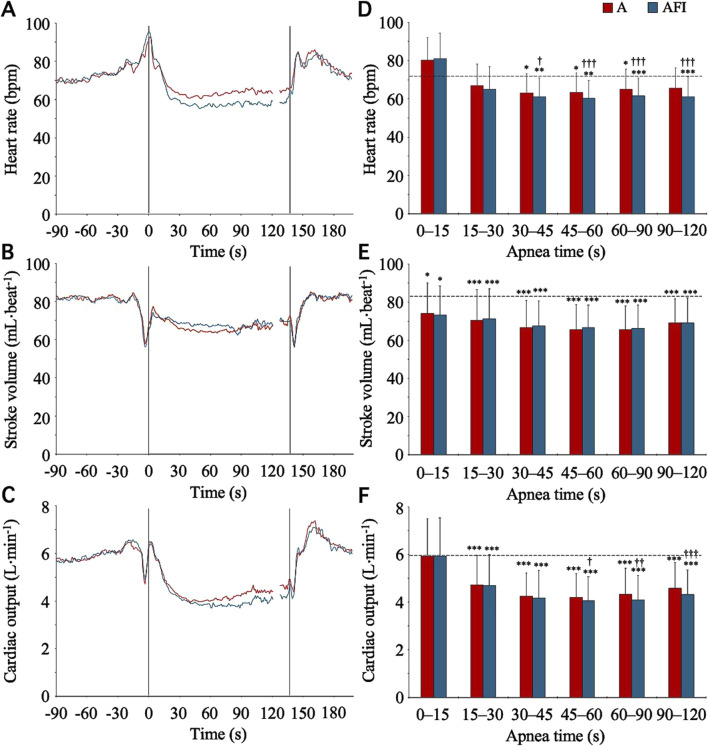
Heart rate, stroke volume, and cardiac output in association with apnea, either without (A) or with face immersion (AFI) in cold water (n = 18, group II). Panel 3 **(A–C)** shows the means of each variable from before apneas (-90–0 s), during the first 120 s of apneas (0–120 s), during the last 10 s of apneas (127–137 s), and the first 60 s after apneas (137–197 s). Vertical lines indicate the start and end of apneas. Breaks in the lines reflect the fact that apnea durations varied among the participants, and the position of the end of tests in the graphs has been adjusted for each participant to match the average duration of the apneas (137 s). Error bars have been omitted for clarity. Panel 3 **(D–F)** shows the means (SD) of each variable during the specified apneic periods (n = 17 for the period 90–120 s due to shorter apnea time in one participant). The horizontal, dashed lines represent the corresponding baseline levels. ^*^ *p* < 0.05, ^**^ *p* < 0.01, and ^***^ *p* < 0.001 compared to baseline. ^†^ *p* < 0.05, ^††^ *p* < 0.01, and ^†††^ *p* < 0.001 compared to apnea without face immersion.

The baseline, eupneic HR, SV, and CO before apnea was 71.4 (11.0) bpm, 84.1 (16.5) mL⋅beat^-1^, and 6.0 (1.4) L⋅min^-1^, respectively. During apneas, the HR of the participants in group II followed a time course that was similar to the time course of the HR of the participants in group I (Figures 3A, D). Starting from a level above baseline for the initial 0–15 s of apnea, the HR gradually decreased, reaching a stable level below the eupneic baseline after 30 s of apnea. During the period 30–120 s into apnea, the HR was reduced from baseline in both apnea without face immersion (90 (10) % of baseline, *p* < 0.001 vs. baseline) and apnea with face immersion (85 (9) % of baseline, *p* < 0.001 vs. baseline, *p* < 0.001 between apneic conditions). Contrary to the HR, the SV never increased above baseline during apneas (Figures 3B, E). Instead, during both apnea without face immersion and apnea with face immersion, the SV was steadily reduced from baseline (*p* < 0.05 vs. baseline), without a difference between apneic conditions. During the period 30–120 s into apnea, the SV was reduced to 80 (7) % of baseline during apnea without face immersion (*p* < 0.001 vs. baseline), and to 81 (6) % of baseline during apnea with face immersion (*p* < 0.001 vs. baseline). With the changes in HR and SV, the CO displayed a time course for changes during apnea that was largely similar to the changes observed for HR (Figures 3C, F). From the baseline level, the CO was gradually reduced for the first 30 s of apnea, after which it remained stable below the baseline level (*p* < 0.001 vs. baseline). During the period 30–120 s into apnea, the CO was reduced in both apnea without face immersion (73 (11) % of baseline, *p* < 0.001 vs. baseline) and apnea with face immersion (69 (10) % of baseline, *p* < 0.001 vs. baseline, *p* < 0.01 between apneic conditions).

**FIGURE 4:**
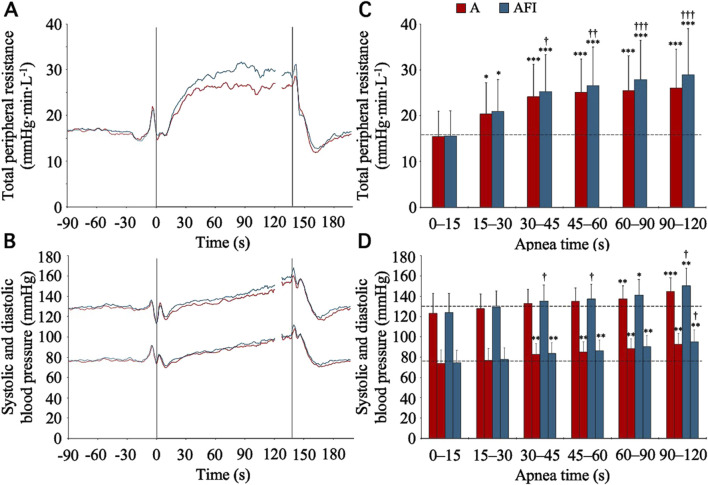
Total peripheral resistance, systolic blood pressure, and diastolic blood pressure in association with apnea, either without (A) or with face immersion (AFI) in cold water (n = 18, group II). Panel 4 **(A, B)** shows the means of each variable from before apneas (-90–0 s), during the first 120 s of apneas (0–120 s), during the last 10 s of apneas (127–137 s), and the first 60 s after apneas (137–197 s). Vertical lines indicate the start and end of apneas. Breaks in the lines reflect the fact that apnea durations varied among the participants, and the position of the end of tests in the graphs has been adjusted for each participant to match the average duration of the apneas (137 s). Error bars have been omitted for clarity. Panel 4 **(C, D)** shows the means (SD) of each variable during the specified apneic periods (n = 17 for the period 90–120 s due to shorter apnea time in one participant). The horizontal, dashed lines represent the corresponding baseline levels. ^*^ *p* < 0.05, ^**^ *p* < 0.01, and ^***^ *p* < 0.001 compared to baseline. ^†^ *p* < 0.05, ^††^ *p* < 0.01, and ^†††^ *p* < 0.001 compared to apnea without face immersion.

Alongside the cardiac responses to apnea, there were vascular responses. During the baseline period, the TPR was 15.9 (4.5) mmHg⋅min⋅L^-1^. From this level, from 15 s into apnea and onwards, the TPR increased (*p* < 0.05 vs. baseline), during both apnea without and with face immersion (Figures 4A, C). During the period 30–120 s into apnea, the TPR was higher during apnea with face immersion (174 (24) % of baseline, *p* < 0.001 vs. baseline) than during apnea without face immersion (160 (20) % of baseline, *p* < 0.001 vs. baseline, *p* < 0.001 between apneic conditions). The baseline levels for systolic and diastolic blood pressures were 130.4 (13.1) and 75.8 (9.5) mmHg, respectively. With the increases in TPR, there were gradual increases in systolic and diastolic blood pressures during apnea (Figures 4B, D), although the blood pressures were not above the baseline levels for the initial 30 s of apnea. From 30 s into apnea and onwards, there were increases in diastolic blood pressure, during both apnea with and without face immersion, while the increase in systolic blood pressure was not evident until 60 s into apnea. On average during the period 30–120 s into apnea, the diastolic blood pressure was 116 (7) % of baseline during apnea without face immersion (*p* < 0.001 vs. baseline) and 118 (12) % of baseline during apnea with face immersion (*p* < 0.001 vs. baseline), without a difference between apneic conditions. During the same period, the systolic blood pressure was 106 (5) % of baseline during apnea without face immersion (*p* < 0.001 vs. baseline) and 109 (9) % of baseline during apnea with face immersion (*p* < 0.001 vs. baseline), also without a difference between apneic conditions.

**FIGURE 5:**
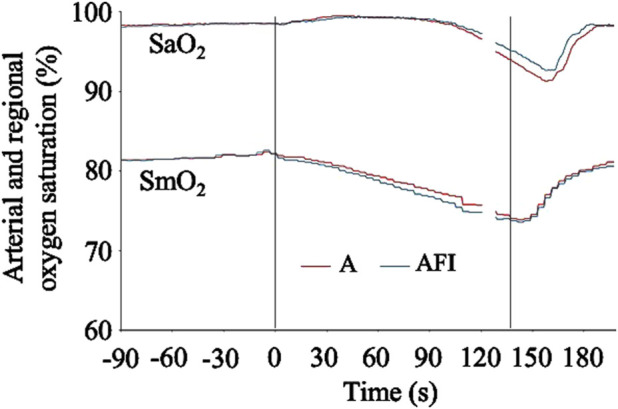
Arterial hemoglobin oxygen saturation (SaO_2_) and regional, deltoid muscle oxygen saturation (SmO_2_) in association with apnea, either without (A) or with face immersion (AFI) in cold water (n = 18, group II). The lines show the means of each variable from before apneas (-90–0 s), during the first 120 s of apneas (0–120 s), during the last 10 s of apneas (127–137 s), and the first 60 s after apneas (137–197 s). Vertical lines indicate the start and end of apneas. Breaks in the lines reflect the fact that apnea durations varied among the participants, and the position of the end of tests in the graphs has been adjusted for each participant to match the average duration of the apneas (137 s). Error bars have been omitted for clarity.

The baseline, eupneic SaO_2_ and SmO_2_ before apnea was 98.1 (0.8) % and 81.5 (7.1) %, respectively. The SaO_2_ was at, or slightly above, the baseline level during the initial 90 s of apnea, after which a gradual decrease in SaO_2_ became evident (Figure 5). There was a greater arterial desaturation associated with the apnea without face immersion. On average, the individual nadir SaO_2_ was 87.2 (9.5) % after apnea without face immersion (*p* < 0.001 vs. baseline), and 88.7 (8.2) % after apnea with face immersion (*p* < 0.001 vs. baseline, *p* = 0.01 vs. apnea without face immersion). The SmO_2_ began to fall immediately upon initiation of apnea (Figure 5). On average during the period 30–120 s into apnea, the SmO_2_ was 78.6 (6.4) % during apnea without face immersion (*p* < 0.001 vs. baseline) and 77.9 (6.4) % during apnea with face immersion (*p* < 0.001 vs. baseline, *p* = 0.04 vs. apnea without face immersion).

### 3.3 Comparisons between group I and group II

For variables recorded and analyzed in equivalent ways, the results of the participants of group I and group II were compared. The baseline, eupneic HR did not differ between group I and group II. Also, the relative reduction in HR during the period 30–120 s into apnea did not differ between participants of group I and group II, neither during apnea without face immersion, nor during apnea with face immersion. Likewise, the time-averaged, apneic pulmonary O_2_ uptake did not differ between group I and group II. However, the time-averaged, apneic pulmonary CO_2_ elimination was slightly lower among the participants of group II compared to the participants of group I (*p* = 0.02 for apnea without face immersion, *p* = 0.04 for apnea with face immersion).

## 4 Discussion

This study examines the effects of apnea with and without cold-water face immersion on the time courses for pulmonary O_2_ uptake and cardiovascular responses in resting humans. The primary findings demonstrate that the pulmonary O_2_ uptake was gradually reduced during the initial 30–45 s of apnea, reaching a level that was below eupneic baseline, and that this time course was similar to the time courses for the cardiovascular responses, especially the apnea-induced reductions in HR and CO. Furthermore, the reductions in pulmonary O_2_ uptake, HR, and CO were greater during apnea with cold-water face immersion than during apnea without face immersion, while the reduction in SV was unaffected by face immersion. The cardiovascular responses seemed to reduce the depletion of the pulmonary O_2_ store during apnea, while the peripheral O_2_ stores, in the present study represented by the SmO_2_, were depleted faster and to a greater extent. Taken together, our study supports previous observations concerning the human diving response’s effects on the pulmonary and peripheral venous O_2_ stores (Lindholm and Linnarsson, 2002; Schagatay et al., 2007; Andersson et al., 2008), and suggests that the more pronounced the cardiovascular adjustments, the longer until severe arterial hypoxemia will develop.

In the present study, apnea with and without cold-water face immersion initiated the typical cardiovascular adjustments that are collectively called the human diving response (Fitz-Clarke, 2018). The reductions in HR and CO, as well as the increase in TPR, were augmented with face immersion, in accordance with previous observations (Kawakami et al., 1967; Sterba and Lundgren, 1988; Cherouveim et al., 2013). At the same time, the apneic changes in SV and arterial blood pressure were unaffected by face immersion. The time courses for the cardiovascular changes in the present study were similar to time courses previously reported (Perini et al., 2008; Perini et al., 2010; Taboni et al., 2019). I.e., from 30 s and onwards during apnea, the cardiac responses were relatively stable, while the arterial blood pressure continued to increase during the apneic period. The cardiovascular responses had discernible effects on the time courses of changes in pulmonary, arterial, and peripheral tissue O_2_ stores.

The pulmonary gas exchange reached a relatively stable, sub-eupneic level within 30–45 s of apnea, essentially following the time course of changes in HR and CO. It has been shown that the pulmonary gas exchange is reduced during apnea compared to eupneic control in resting humans (Linér and Linnarsson, 1994; Wein et al., 2007; Andersson et al., 2008), and that this reduction can be attributed to the cardiovascular diving response. However, the resemblance of the time course for apneic pulmonary gas exchange with the cardiovascular adjustments comprising the human diving response has not been demonstrated previously in resting humans. In a pioneering work by Lanphier and Rahn (1963), albeit including only four subjects, they suggested that all or part of the reduction in alveolar gas exchange during apnea could be attributed to a decrease in CO. Ferretti et al. (1991) studied apneas at rest in three elite breath-hold divers of the same family and nine non-diver control subjects. They reported that the apneic O_2_ uptake was gradually reduced from eupneic control in the elite breath-hold divers, but not in the control subjects. Linér et al. (1993), studying apneas at the surface and during compression to 20 m in a hyperbaric chamber in five subjects, reported no significant changes in apneic O_2_ uptake during surface apneas, while the CO_2_ elimination was gradually reduced with a time course similar to that of the present study. It should be noted that neither of these studies included recordings of cardiovascular changes and hence no direct correlation of changes in pulmonary gas exchange to cardiovascular responses could be reported. Nevertheless, based on previous studies of cardiovascular responses to apnea, Lanphier and Rahn (1963), Ferretti et al. (1991), and Linér et al. (1993) suggested that the observed changes in gas exchange were partly attributable to changes in CO and peripheral blood flow. Studying apneas performed during steady-state dynamic leg exercise, Lindholm and Linnarsson (2002) discussed how gradual reductions in pulmonary O_2_ uptake during apnea were related to observed reductions in HR and assumed reductions in mixed venous O_2_ saturation. These latter observations from exercising subjects are in accordance with the findings of the present study with resting subjects, and together illustrate how the cardiovascular changes of the diving response contribute to the reduced pulmonary gas exchange during apnea.

In the present study (group I), the apneic pulmonary O_2_ uptake during the entire 2-min apnea without or with face immersion in cold water was reduced by 12% and 16%, respectively, compared to baseline, eupneic pulmonary O_2_ uptake. These reductions are slightly smaller than the 19% and 23% reductions reported in a similar study performed in our lab (Andersson et al., 2008), using a similar protocol. The difference in apnea times between the two studies may largely explain the smaller reductions in pulmonary O_2_ uptake in the present study. In the previous study, the average apnea times were 184 s, more than 1 min longer than the longest apneas in group I in the present study. When shorter apneas are considered, the impact of the relatively high pulmonary O_2_ uptake during the initial 30 s of apnea on the time-averaged apneic O_2_ uptake becomes more prominent. In the present study, this is illustrated by the observation that during the period 30–120 s into apnea without and with face immersion, when the reduction in gas exchange had stabilized, the O_2_ uptake was reduced by 29% and 32%, respectively, from eupneic baseline. This impact of apneic duration on the time-averaged apneic pulmonary O_2_ uptake, causing the pulmonary O_2_ uptake to be reduced to a greater extent the longer the apnea, may partly explain why earlier studies (e.g., Sterba and Lundgren, 1988) have not reported a reduction in apneic pulmonary O_2_ uptake compared to eupneic baseline. I.e., if too short apneas have been used, the reduction in pulmonary O_2_ uptake that occurs after some 30 s of apnea may have passed by undetected.

Of interest is the close association between changes in CO and changes in pulmonary O_2_ uptake. During the period 30–120 s into apnea, the CO was reduced by 27% and 31% compared to baseline, during apnea without and with face immersion, respectively. This is remarkably similar to the 29% and 32% reductions in pulmonary O_2_ uptake that we report for the same apneic period. This observation aligns with the notion that apnea-induced changes in pulmonary O_2_ uptake is largely dependent on changes in CO during apnea, reducing the uptake secondary to reduced pulmonary perfusion (Persson et al., 2023). Several studies employing different methods have shown that an augmented diving response is associated with a reduced rate of arterial desaturation and a reduced rate of depletion of the pulmonary O_2_ store during apnea at both rest and exercise (e.g., Andersson and Schagatay, 1998; Lindholm et al., 1999; Stewart et al., 2005; Andersson et al., 2008; Andersson and Evaggelidis, 2009; Marabotti et al., 2013). It is the cardiovascular adjustments during apnea–including a decrease in CO, a redistribution of systemic blood flow, and a peripheralization of venous blood volume–that bring about this O_2_-conserving effect. This effect is defined here as a temporary reduction in pulmonary O_2_ uptake, a preservation of the pulmonary O_2_ store, and a reduced rate of arterial desaturation. However, the preservation of the pulmonary O_2_ store occurs at the expense of the peripheral venous O_2_ stores. The increased TPR limits peripheral O_2_ delivery and causes a widening of the arterial-to-venous difference in O_2_ content, in the present study indicated by the immediate reduction in SmO_2_ while the SaO_2_ is maintained. Even though the arterial-to-venous difference in O_2_ content in the peripheral tissues increases due to the reduced blood flow and this intuitively may seem to argue against an O_2_-conserving effect (Lin and Hong, 1996), this will contribute to the preservation of the pulmonary O_2_ store because of a simultaneous prolongation of the turnover time of the peripheral venous blood (Linér and Linnarsson, 1994; Andersson et al., 2004). This explains why skeletal muscle desaturation occurs earlier and to a greater extent than the cerebral desaturation during apnea (Palada et al., 2007b; Persson et al., 2023), having the potential to allow longer breath-holding times (Schagatay and Andersson, 1998). With a more pronounced diving response, the time for reaching hypoxic levels that threatens the function of the heart and the brain during apnea is delayed. In the present study the reduced pulmonary O_2_ uptake and higher P_ET_O_2_ at the end of apnea with face immersion, together with higher SaO_2_, supports the idea of preservation of the pulmonary O_2_ store with an augmented diving response. The greater reduction in SmO_2_ during apnea with face immersion indicates a greater depletion of peripheral venous O_2_ stores compared to during apnea without face immersion.

The longest apneas in the present study were 120 s in group I and on average 156 s in group II. These apnea times are considerably shorter than the breath-holding times regularly performed by experienced breath-hold divers, whose competitive static apnea times are often longer than 5 minutes, and the current world record, which is 11 min 35 s (Fitz-Clarke, 2018). Even though the turnover time of desaturated peripheral venous blood is prolonged during apnea (Linér and Linnarsson, 1994), this desaturated blood most likely returns to the central circulation during later phases of a longer apnea. This would have the potential to increase pulmonary O_2_ uptake later into apnea. However, at the same time the reduced alveolar PO_2_ will simultaneously decrease the alveolo-capillary O_2_ difference (Lanphier and Rahn, 1963; Hong et al., 1971), thereby reducing the O_2_ uptake. Thus, from the present study it is not possible to draw any conclusions regarding the temporal changes in pulmonary gas exchange in later phases of longer apneas, such as those performed by experienced, competitive breath-hold divers.

As expected, there was a striking reduction in pulmonary CO_2_ elimination during apnea compared to the eupneic baseline in the present study, a reduction that was much larger that the reduction in pulmonary O_2_ uptake. This finding is in accordance with earlier observations (Lanphier and Rahn, 1963; Hong et al., 1971; Linér et al., 1993). There was no difference in pulmonary CO_2_ elimination between apneas without and with face immersion, indicating that the reduction is affected to a larger extent by other factors than the cardiovascular adjustments. The alveolar PCO_2_ will increase with apnea, getting closer to and possibly exceeding the mixed-venous PCO_2_. Thus, as apnea proceeds, the diffusion gradient for CO_2_ is gradually reduced and may eventually be reversed compared to in the eupneic condition, leading to a reduced, and possibly reversed, pulmonary CO_2_ elimination (Hong et al., 1971; Linér et al., 1993).

## 5 Conclusion

The present study shows that the temporal changes of the cardiovascular diving response are associated with parallel temporal changes in pulmonary gas exchange, especially pulmonary O_2_ uptake, supporting the view that the human diving response has an O_2_-conserving effect by a reduction in CO and a redistribution of peripheral blood flow. The pulmonary O_2_ uptake is gradually reduced during apnea, with changes from control in agreement with simultaneous changes in HR and CO. As the cardiovascular diving response is augmented by cold-water face immersion during apnea, the reduction in pulmonary O_2_ uptake was greater during apnea with face immersion. Thus, we conclude that the central, pulmonary O_2_ store is preserved with an augmented diving response, at the expense of peripheral venous O_2_ stores, in the present study represented by the deltoid muscle O_2_ saturation. Any temporal changes in pulmonary gas exchange in longer apneas than those included in the present study, and their relationship to cardiovascular changes, remain to be established.

## Data Availability

The raw data supporting the conclusions of this article will be made available by the authors, without undue reservation.
